# Supplementary material for: Role of the imprinted allele of the Cdkn1c gene in mouse neocortical development
Source: Sci Rep. 2020 Feb 5;10:1884. doi: 10.1038/s41598-020-58629-9 (PMC7002495; doi:10.1038/s41598-020-58629-9)
Supplement: Supplementary file 1 — Supplementary Information. [file 41598_2020_58629_MOESM1_ESM.pdf]

Supplementary Information

Role of the imprinted allele of the *Cdkn1c* gene in mouse neocortical development

Yui Imaizumi[1], Shohei Furutachi[1,2], Tomoyuki Watanabe[1], Hiroaki Miya[1], Daichi Kawaguchi[1]* & Yukiko Gotoh[1,3]*

[1]Graduate School of Pharmaceutical Sciences, The University of Tokyo, Tokyo 113-0033, Japan
[2]Sainsbury Wellcome Centre for Neural Circuits and Behaviour, University College London, London W1T 4JG, UK
[3]International Research Center for Neurointelligence (WPI-IRCN), The University of Tokyo, Tokyo 113-0033, Japan.

*Correspondence and requests for materials should be addressed to Y.G. (email: ygotoh@mol.f.u-tokyo.ac.jp) and D. K. (email: dkawaguchi@mol.f.u-tokyo.ac.jp).

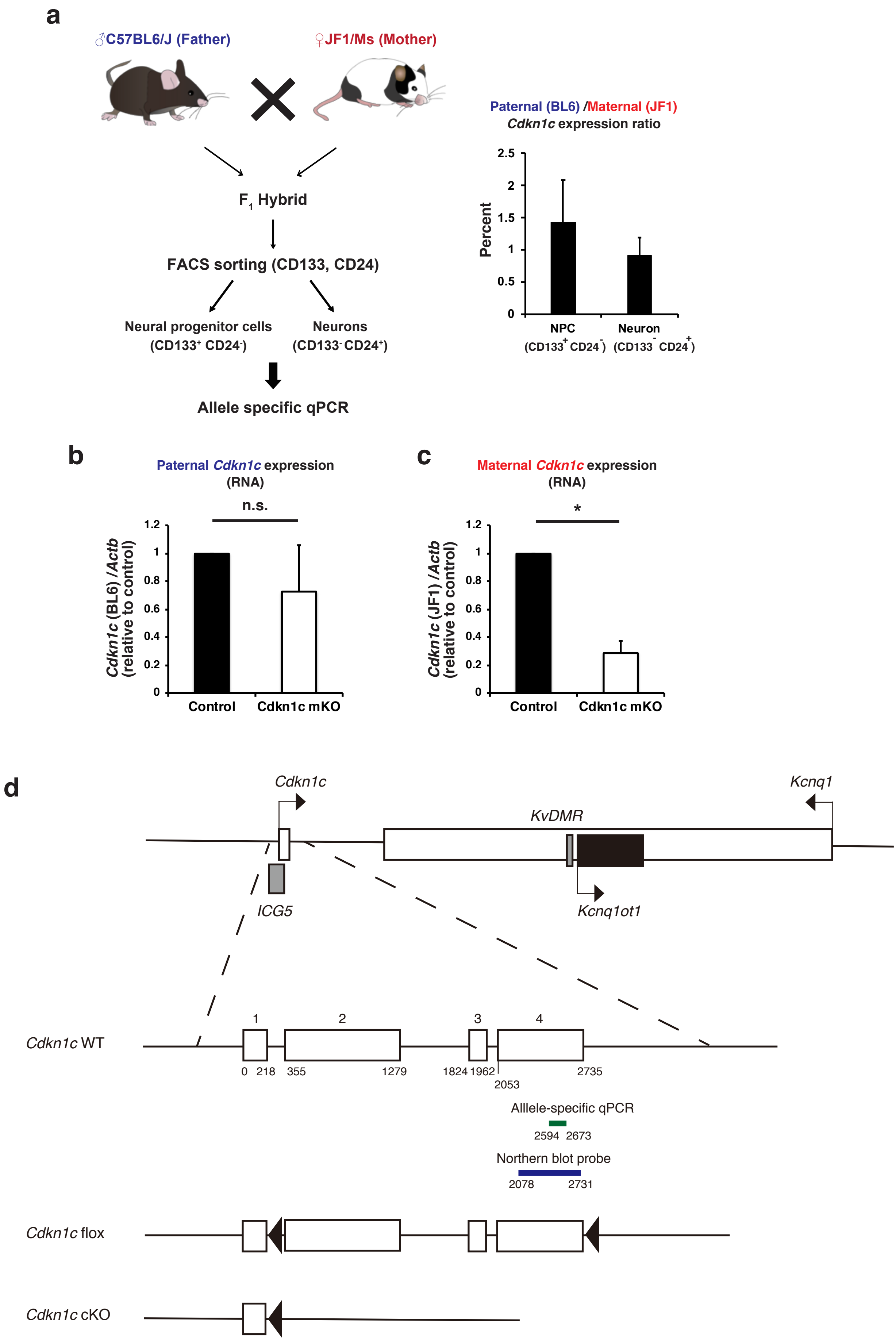
a
♂C57BL6/J (Father)
♀JF1/Ms (Mother)
F1 Hybrid
FACS sorting (CD133, CD24)
Neural progenitor cells
(CD133+ CD24-)
Neurons
(CD133- CD24+)
Allele specific qPCR
Paternal (BL6) /Maternal (JF1)
Cdkn1c expression ratio
Percent
NPC
(CD133+ CD24-)
Neuron
(CD133- CD24+)
b
Paternal Cdkn1c expression
(RNA)
n.s.
Cdkn1c (BL6) /Actb
(relative to control)
Control
Cdkn1c mKO
c
Maternal Cdkn1c expression
(RNA)
*
Cdkn1c (JF1) /Actb
(relative to control)
Control
Cdkn1c mKO
d
Cdkn1c
Kcnq1
KvDMR
ICG5
Kcnq1ot1
Cdkn1c WT
1
2
3
4
0
218
355
1279
1824
1962
2053
2735
Alllele-specific qPCR
2594
2673
Northern blot probe
2078
2731
Cdkn1c flox
Cdkn1c cKO

Supplementary Figure 1. Detection of *Cdkn1c* mRNA expression from each allele and the schematic representation of *Cdkn1c* floxed allele.

(a) The ratio of paternal to maternal *Cdkn1c* mRNA expression in NPCs and in neurons was determined by allele specific qPCR under the reciprocal hybrid (BL6 male and JF1 female) genetic background at E16. FACS was used to isolate NPCs ($CD133^{+}$ $CD24^{-}$ population) and neurons ($CD133^{-}$ $CD24^{+}$ population).

(b)(c) Allele specific qPCR of *Cdkn1c* mRNA in the neocortex isolated from control (*Cdkn1c* $^{mat\ flox\ (BL6)/+(JF1)}$) and hybrid maternal *Cdkn1c* cKO (Nestin-Cre; *Cdkn1c* $^{mat\ flox\ (BL6)/+(JF1)}$) mice at E16. *Cdkn1c* mRNA expression from each allele was normalized to *β-actin*. Data are expressed relative to the corresponding value for control mice.

(d) Schematic representations of the wild-type (WT) mouse *Cdkn1c* allele, the floxed *Cdkn1c* allele (Flox), and the *Cdkn1c* allele after removal of exons 2 to 4 by Cre recombinase. LoxP sites are indicated as triangles, differentially methylated regions (DMRs) and exons are shown as gray boxes and white boxes, respectively.

Data are mean+s.e.m from three independent experiments.

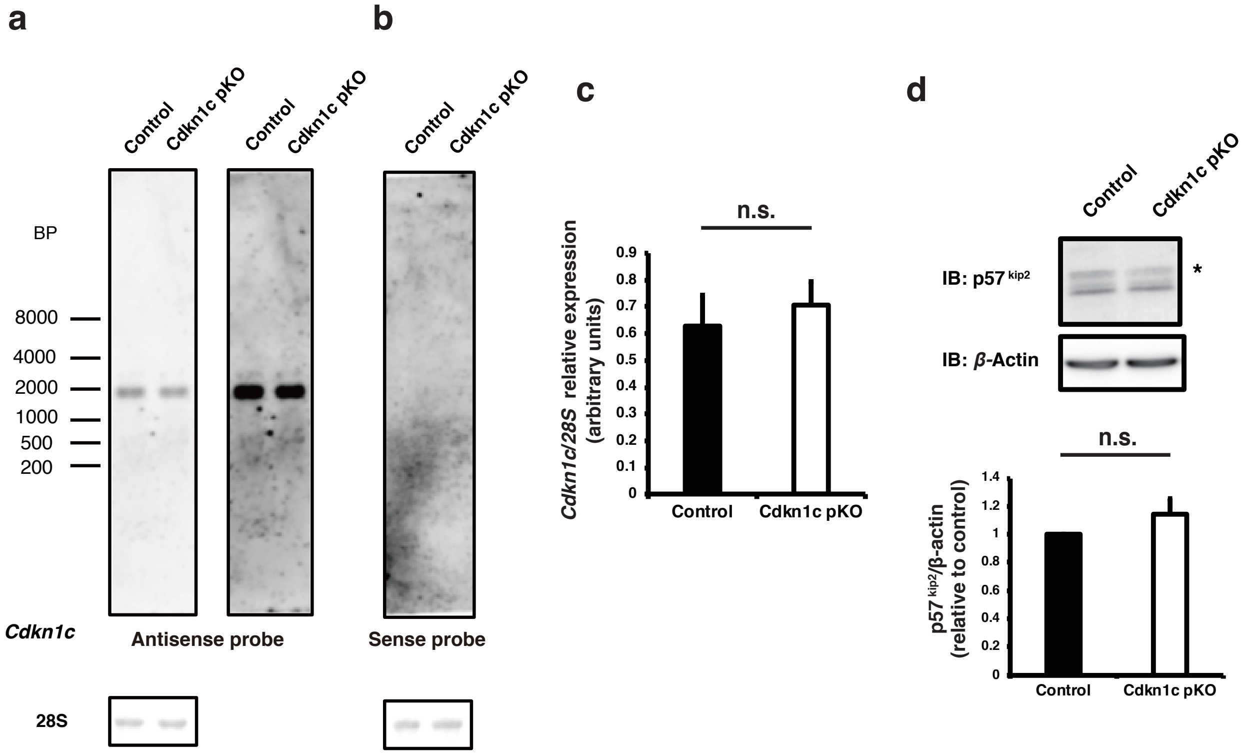

Supplementary Figure 2. Detection of *Cdkn1c* mRNA expression from the sense strand and p57 ^{kip2} protein expression.

(a-b) Detection of *Cdkn1c* mRNA expression by Northern hybridization using antisense (**a**) and sense (**b**) probes in the neocortex of *Cdkn1c* paternal cKO and control mice at P0. The signal obtained with 28S is shown as a loading control (bottom). The exposure time is different between the left and right panels in (**a**).

(c) Quantification of signal intensities in (**a**). (n = 4-7 pups for each genotype). *Cdkn1c* mRNA expression was normalized to 28S expression. Data are mean+s.e.m. Unpaired two-tailed Student's t-test.

(d) Immunoblotting of p57 ^{kip2} and β-actin in the neocortical lysates of paternal *Cdkn1c* cKO and control mice at P0 are shown in the upper panels. Asterisk indicates a non-specific band. Quantification of the data is shown in the lower panel. Data are mean+s.e.m from three independent experiments. Paired two-tailed Student's t-test.

n.s., not significant.

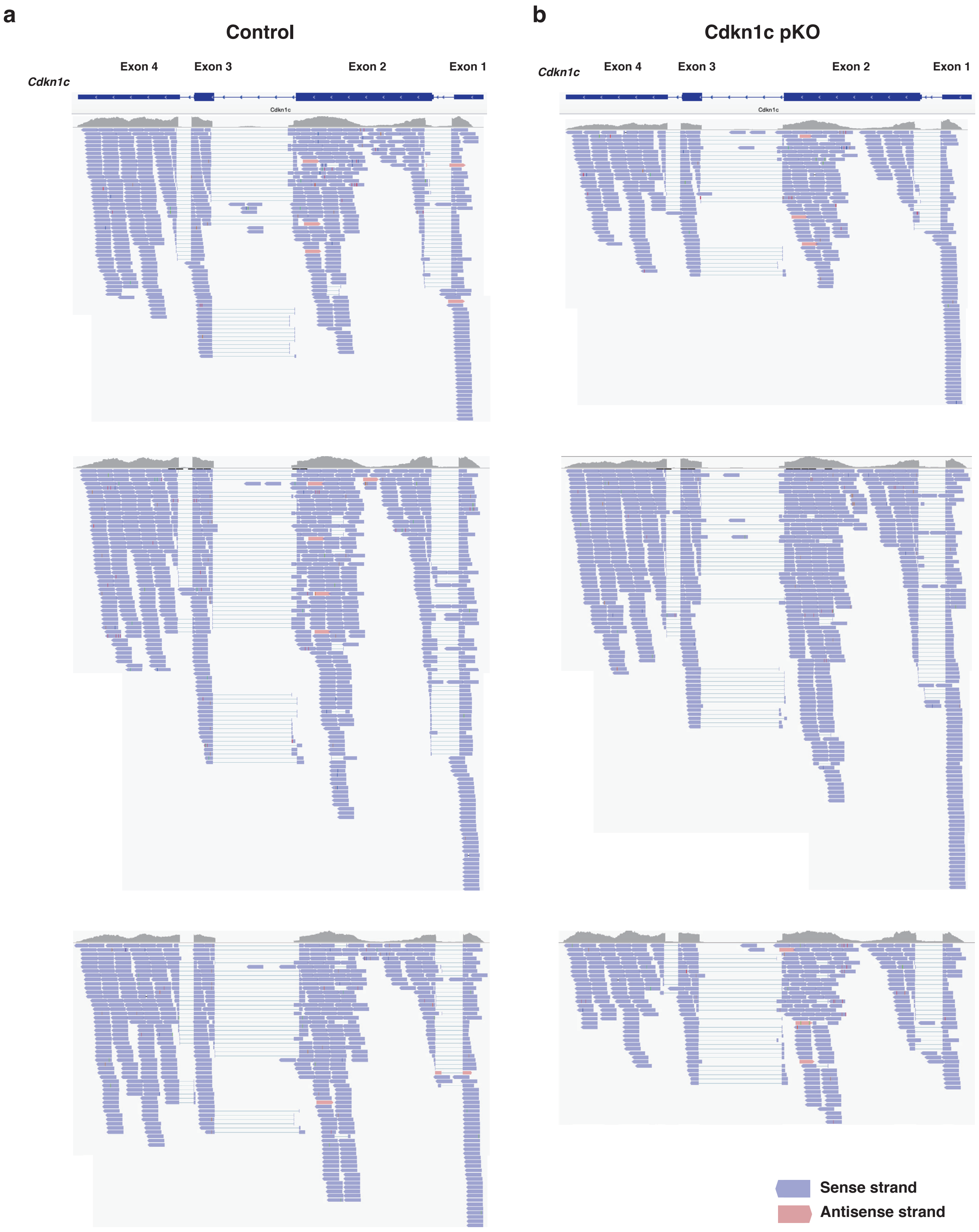
a
Control
Exon 4
Exon 3
Exon 2
Exon 1
Cdkn1c
b
Cdkn1c pKO
Exon 4
Exon 3
Exon 2
Exon 1
Cdkn1c
Sense strand
Antisense strand

Supplementary Figure 3. Direction (strand)–sensitive RNA-sequencing analysis.

(a-b) Integrative Genomics Viewer (IGV) screenshots for the *Cdkn1c* locus in the neocortex of control (**a**) and paternal *Cdkn1c* cKO (**b**) mice at E16 are shown. Each sequencing read of sense and antisense strands is shown in blue and red boxes, respectively.

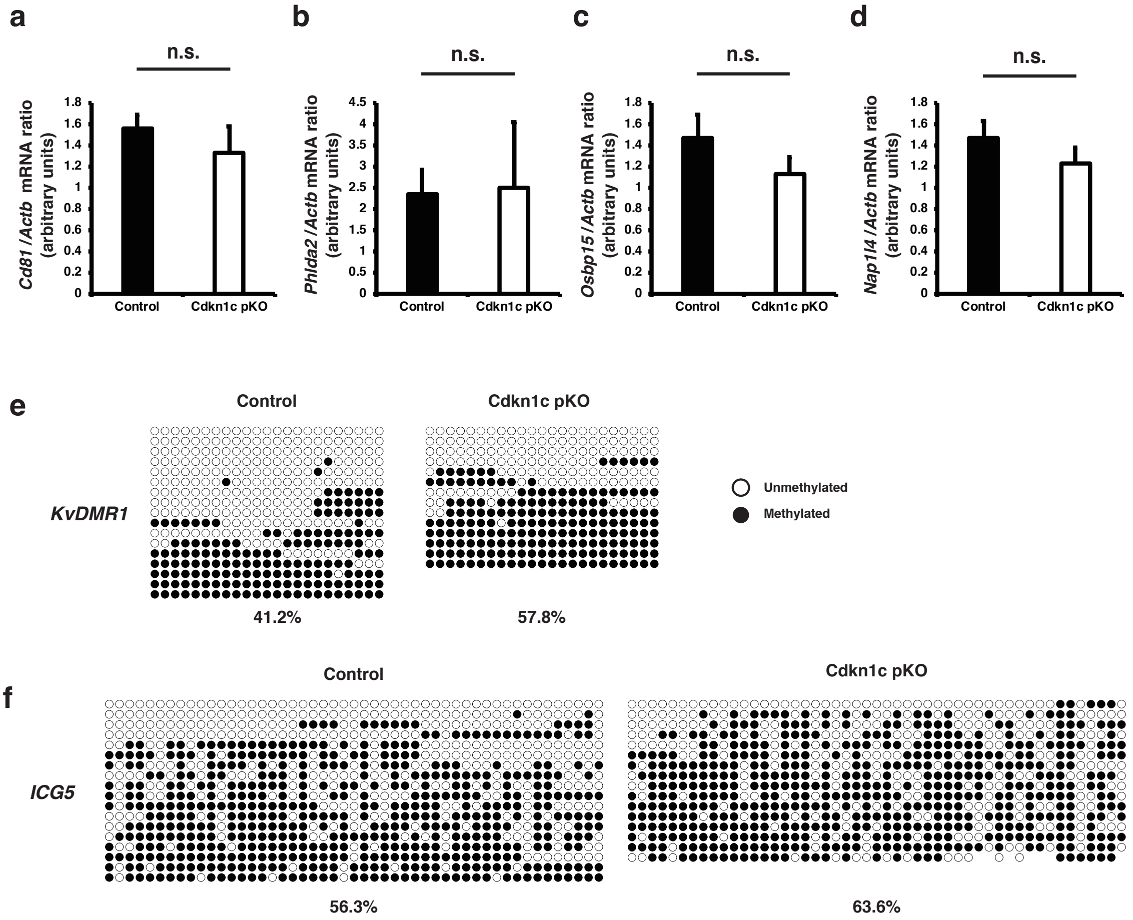

Supplementary Figure 4. Paternal *Cdkn1c* conditional deletion did not largely affect the levels of gene expression or DNA methylation at differentially methylated regions nearby the *Cdkn1c* locus.

(a-d) qPCR of mRNA expression of the genes nearby *Cdkn1c* in the neocortex isolated from control and paternal Cdkn1c cKO mice at P0. mRNA expression was normalized to *β-actin*. (n=4-8 for each genotype). Data are mean+s.e.m. Unpaired two-tailed Student's t-test. n.s., not significant.

(e-f) DNA methylation analysis of KvDMR and ICG5 in the brain of control and paternal *Cdkn1c* cKO mice at E16. Open and closed circles represent unmethylated and methylated CpGs, respectively. Each row represents one individual clone of amplified PCR products.

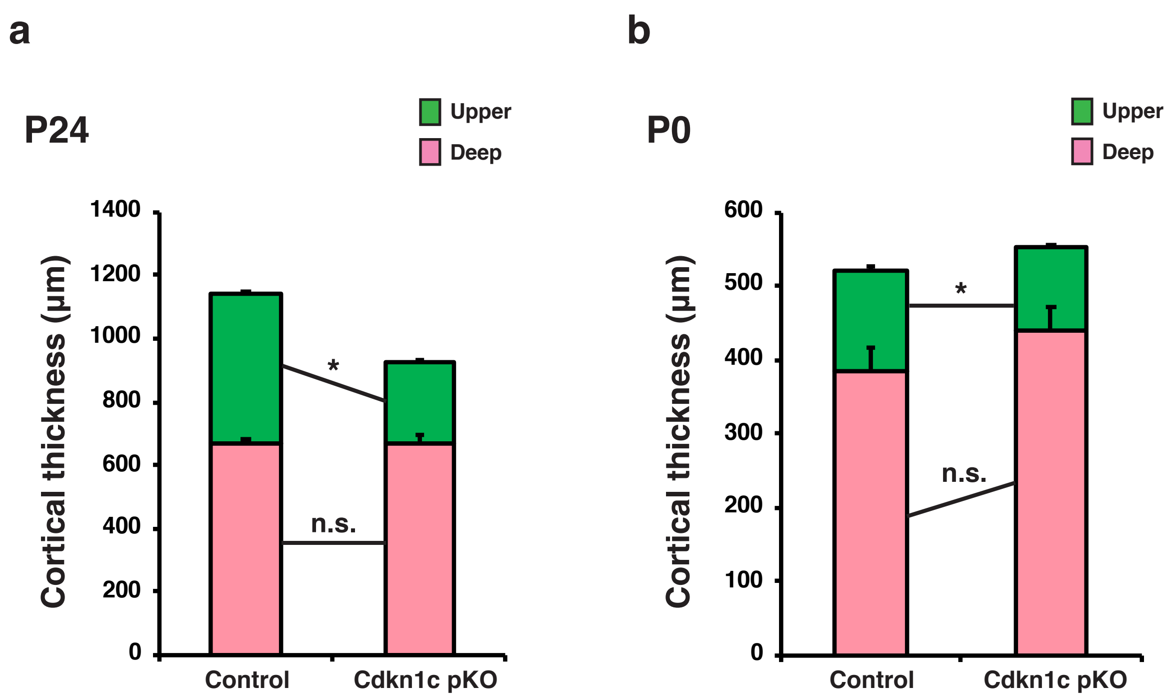

Supplementary Figure 5. CNS-specific deletion of the *Cdkn1c* paternal allele reduced the thickness of upper layers.

(a-b) Cortical thickness of upper layer neurons (Cux1^{+}) and deep layer neurons (Ctip2^{+}) in the somatosensory area was quantified at P24 (**a**) and P0 (**b**)

Data are mean+s.e.m. Unpaired two-tailed Student's t-test; n=3 mice for each genotype in (**a**), n=5 mice for each genotype in (**b**); ****P*** <0.05. n.s., not significant.

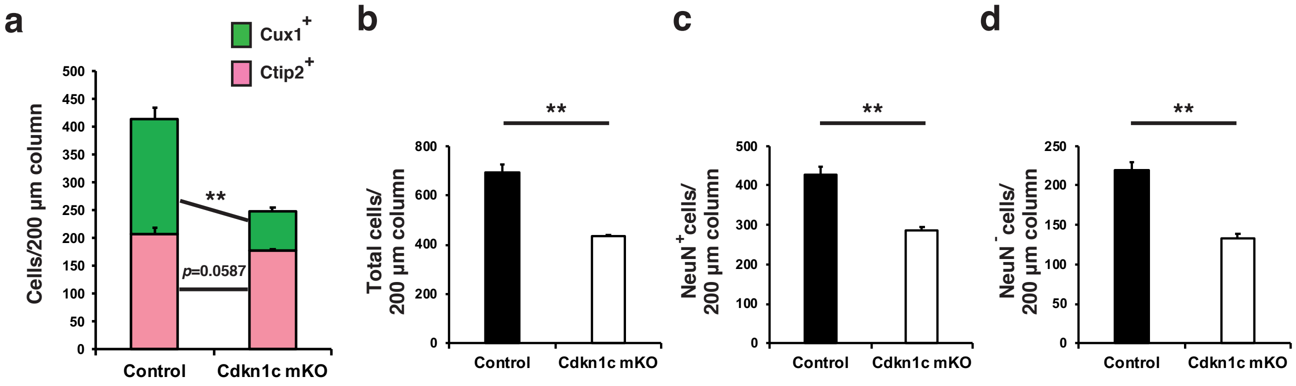

Supplementary Figure 6. Characterization of the neocortical phenotype induced by CNS-specific deletion of the maternal *Cdkn1c* allele.

(a) Quantitative analysis of cells positive for Cux1 and Ctip2 per area within 200 μm wide bins.

(b-d) The number of total cells (Hoechst^{+}) (**b**), neurons (NeuN^{+}) (**c**) and non-neuronal cells (NeuN^{-}) (**d**) per 200 μm wide bins in the neocortical area were quantified.

Data are mean+s.e.m. Unpaired two-tailed Student's t-test; n=3 mice for each genotype, ***P* <0.01.

a

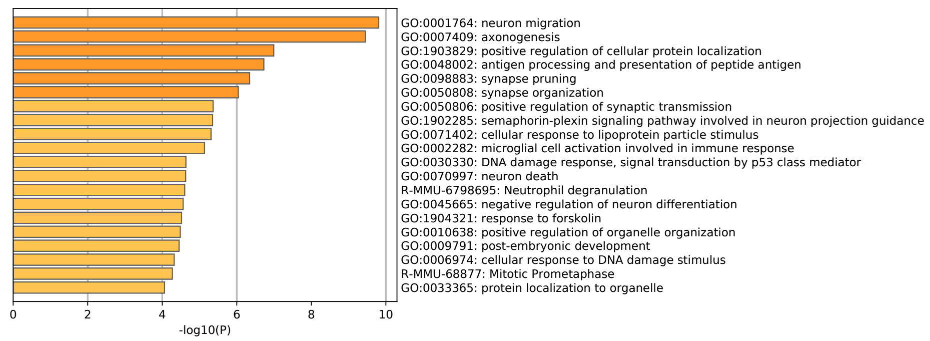

b

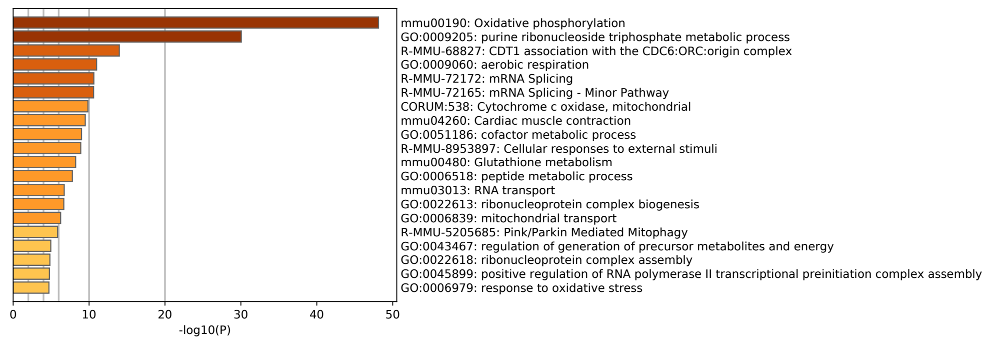

Supplementary Figure 7. GO biological process analysis of dysregulated genes by the paternal *Cdkn1c* deletion.

(a-b) The GO terms associated with significantly upregulated (**a**) and downregulated (**b**) genes by paternal *Cdkn1c* deletion are shown. Adjusted P values are based on Metascape software[44].
